# Effect of affective feedback and competitiveness on performance and the psychological experience of exercise within a virtual reality environment

**DOI:** 10.1371/journal.pone.0268460

**Published:** 2022-06-08

**Authors:** Nicole Trewick, David L. Neumann, Kyra Hamilton

**Affiliations:** 1 School of Applied Psychology, Griffith University, Gold Coast, Queensland, Australia; 2 Menzies Health Institute Queensland, Griffith University, Gold Coast, Queensland, Australia; University of Pavia, ITALY

## Abstract

Exercise is beneficial for physical and psychological health, yet the majority of Australian adults are not sufficiently active to gain health benefits. Novel methods are needed to enhance the experience of exercise and ultimately exercise participation. The present study examined performance and psychological experiences during a (non-immersive) virtual reality cycling task that incorporated affective feedback. Female participants (*N* = 137, university students) received either positive, negative, or neutral virtual feedback while cycling on a stationary bicycle in a virtual reality laboratory environment under the instruction to maintain at least 70% of their maximal heart rate for as long as possible (or up to 30 minutes). Participants also responded to measures of affect, motivation, enjoyment, and competitiveness. Data were analysed with ANOVA’s performed with feedback groups and trait competitiveness for the psychological and performance dependent measures. Results showed that positive feedback elicited greater interest and enjoyment during the task than neutral and negative feedback. In addition, perceived competence was greater with positive feedback than for neutral and negative feedback in low competitive participants. The type of feedback did not affect performance (cycling persistence, perceived exertion, and effort). The findings indicate the potential importance of providing positive virtual feedback and considering the interaction of individual difference factors, specifically competitiveness, to enhance virtual exercise experiences.

## Introduction

The positive health and wellbeing effects of performing regular exercise are well-documented [1]. Given the benefits, much effort has been invested into campaigns and programs to increase people’s engagement in regular exercise. Yet, global prevalence rates indicate that approximately 30% of adults are not sufficiently active, with higher rates (32.5% to 55%) observed in Australia [2]. New ways to increase participation in exercise are therefore needed so that individuals can achieve the physical and psychological benefits of regular exercise [3]. A novel method that may enhance exercise performance and the psychological experience of exercise is the use virtual reality (VR) technology [4], which allows programming to potentially enhance the performance and psychological experience of exercise for the user. VR may also be a useful method to facilitate continued exercise routines where access to health and fitness centres are limited, such as in pandemics.

Neumann et al. [4] provided a synthesis of prior research on VR-based sport and exercise and developed a model showing the relationship between the components of the system and outcomes. The study found the VR system to consist of task factors (e.g. exertion interface), user factors (e.g. traits), VR environment factors (e.g. VR display), and non VR-environment factors (e.g. location) which influences concurrent and post task physiological, performance, and psychological outcomes. These factors may be used in combination to create a unique exercise experience for the user to enhance the performance and psychological outcomes.

Exercising within a VR environment has the potential to increase physical exertion. For example, research has shown participants physically exert themselves more when cycling in compared to not cycling in a virtual world [5]. Similarly, participants rowed faster across a set time trial when rowing was coupled with VR than without VR [6]. Adherence to exercise has also been shown to increase with the addition of VR [7] while exercising in a virtual gymnasium was found to promote engagement with exercise [8].

Improving performance and positive psychological states during VR-based exercise has also been shown when people are provided with feedback (regarding, for example, goals attained, effort, technique). The provision of feedback during exercise provides individuals with the opportunity to adjust their behaviour in a way to increase performance and reach goals. One mechanism through which feedback is argued to work is message valence; that is, the perceived positivity or negativity of the feedback statement [9]. Positive feedback affirms that the person is likely to achieve the desired goal. It enhances the individual’s performance as well as their motivation to achieve the desired outcome [10, 11]. In contrast, negative feedback undermines an individual’s belief in their ability and reduces their expectations of success [12]. Other perspectives, however, argue that negative feedback is more effective than positive feedback because negative feedback signals to the individual that their goal has not yet been achieved, which may boost goal congruent behaviours [12–14]. Positive feedback, in contrast, will signal that the goal has, or at least has partly, been attained. Therefore, in contrast to positive feedback being effective when it signals commitment, negative feedback is effective when it signals a lack of progress [15].

In exercise contexts, valenced feedback has been found to enhance exercise performance and the psychological experiences of exercise. Stoate et al. [16] found running economy of participants increased when positive statements were given compared to no feedback; negative feedback was not explored. Similarly, Hutchinson et al. [17] found participants’ sustained effort (time) increased with positive social comparative feedback compared to negative or no feedback, while perceived exertion and perceived effort decreased with positive compared to negative and no feedback during a grip strength task. Feedback has also been shown to influence psychological experiences, such as affect and intrinsic motivation within an exercise context. In a running task, positive feedback resulted in greater reported positive affect when assessed after the run compared to no feedback [16]. Similar results were found with visual performance feedback provided during a back squat task [18].

Feedback has also been examined in VR-based exercise contexts. Khaghani et al. [8] examined the effect of valenced virtual feedback delivered according to the completeness of exercise tasks during an 8-week VR home-based plan [8]. Although findings from specific valence of feedback were not reported, comparisons were made between the social group who received feedback and interacted with others in a virtual gym and the control group who exercised individually with no feedback or interaction. Results found training plan adherence and participation were greater for social group participants than the control group. While this study suggests that virtual feedback can enhance outcomes, it is unclear as to whether the feedback or social interaction was the influential factor and how different types of feedback might influence exercise behaviour.

In addition to the nature of the feedback statement, source of feedback and recipient (the exerciser) of the feedback are necessary factors to consider. Examining the source of feedback, Ijsselsteijn et al. [19] investigated whether feedback from a virtual coach could enhance intrinsic exercise motivation when participants cycled in a virtual environment. The virtual coach, who was depicted as an avatar, gave feedback at 1-minute intervals on whether the participant’s heart rate was higher or lower than a target heart rate. The presence of virtual feedback had no effect on cycling speed or overall intrinsic motivation. However, virtual feedback was associated with components of intrinsic motivation including higher perceived value and usefulness, reduced pressure and tension, and reduced perceived control. Although the nature of the feedback may have had an affective component (e.g., “your heart rate is too low, cycle faster”), feedback valence was not systematically manipulated in this study. Feedback valence is argued to be an important consideration due to the differential effect on intrinsic motivation for exercise, and mixed theoretical perspectives for whether negative or positive feedback enhances intrinsic motivation [20–22].

In addition, other research has examined factors related to the recipient of feedback including trait competitiveness. Trait competitiveness has been found to be beneficial for exercise performance and psychological experiences of exercise generally [23, 24], and theories suggest a differential effect of valenced feedback for high and low levels of trait competitiveness [25–29]. Competitive VR exercise scenarios were found to lead to increased performance and motivation; highly competitive compared to low competitive adults increased cycling intensity and power when a competitor (avatar) was introduced [30, 31]. In samples of football athletes, performance orientated [18] and visual feedback [32] resulted in higher ratings of task competitiveness and increased football performances when compared to no feedback. The interplay between the nature of feedback and recipient of feedback however is an underexplored area in the literature.

Taken together, limited prior research suggests that feedback may play a role in performance and psychological experiences of exercise, with positive feedback being potentially more beneficial than negative feedback, although many studies have only compared positive feedback with no feedback leaving a knowledge gap on effects of negative feedback. Furthermore, studies that have examined feedback in VR environments have not considered feedback valence, nor considered individual difference variables such as competitiveness that might moderate the effects of feedback. According to social comparison theory [33], individuals are driven to improve their performance and reduce the difference between their own performance and that of others. In a VR exercise task, feedback valence may be perceived differently between those high in trait competitiveness and those low in trait competitiveness. In particular, negative feedback regarding performance deficits may be motivational for high competitiveness individuals because they want to outperform others and enjoy winning [34, 35]. On the other hand, negative feedback may decrease motivation [36] for individuals low in competitiveness as they prefer cooperation and are not concerned about others being better than them at a given task [34, 35]. To the authors’ knowledge, no study to date has explored these possibilities.

The present study addressed the noted limitations in prior research and extended the current knowledge on feedback effects within exercise and performance contexts. It also extended the knowledge on the role of social facilitation and social value orientation, examining how trait competitiveness might moderate the effect of feedback. Thus, the aim of the present study was to examine how positive feedback might increase exercise performance and positive psychological experiences of exercise relative to negative or neutral feedback, consistent with previous findings in non-VR contexts [37]. The relationship between feedback valence and trait competitiveness was also examined. For participants high in competitiveness, negative feedback was expected to increase performance and psychological benefits relative to neutral feedback. In contrast, positive feedback was expected to increase performance and psychological benefits for participants low in competitiveness because positive feedback may indicate that they are performing well [34, 35].

## Method

### Participants

Participants (*N* = 137, *M*_age_ = 22.65 years, *SD* = 7.52) were university students who participated in exchange for partial course credit (*n* = 105) or entry into a chance to win a gift voucher (*n* = 32). Only females were eligible to participate to avoid performance differences found between males and females, specifically owed to the standardised minimum power output required by apparatus setup and cycling cadence. Inclusion criteria included having the ability to cycle and no medical conditions that prevent exercising. Adult Pre-Exercise Screening Tool [38] was used to identify individuals with medical conditions that may place them at a higher risk of an adverse event when undertaking physical activity or exercise. Participants were required to be categorised as low risk, which corresponded to not requiring medical clearance before engaging in vigorous exercise. No participants were screened out at this stage of the study. Participants were randomly allocated using excel file numbering to either the positive (*n* = 45), negative (*n* = 46), or neutral (*n* = 46) feedback groups. Analyses revealed no significant group differences for age, BMI, physical activity categories, heart rate reserve, exercise thoughts, and baseline positive and negative affect (see Table 1).

**Table 1:** Means (standard deviations) for participant descriptive variables in each feedback condition.

	Feedback Condition		
	Positive	Neutral	Negative
Age (years)	23.41 (8.58)	22.35 (6.80)	22.33 (7.28)
Height (cm)	166.69 (5.93)	166.00 (6.48)	166.78 (5.67)
Weight (kg)	62.15 (10.56)	61.99 (10.10)	62.83 (7.93)
Body Mass Index (BMI)	22.35 (3.59)	22.51 (3.67)	22.64 (3.03)
Moderate Intensity Physical Activity (hours per week)	6.89 (7.14)	7.18 (7.45)	10.40 (13.67)
Vigorous Intensity Physical Activity (hours per week)	3.14 (3.92)	3.28 (4.87)	2.60 (3.59)
Sitting Activity (hours per week)	48.75 (19.12)	54.97 (26.77)	50.36 (18.48)
Heart Rate Reserve	136.98 (6.37)	138.33 (4.76)	138.37 (5.09)
Baseline Negative Affect	1.31 (0.39)	1.41 (0.61)	1.23 (0.26)
Baseline Positive Affect	2.78 (0.73)	2.86 (0.59)	2.88 (0.71)
Exercise Thoughts	2.37 (0.69)	2.19 (0.68)	2.40 (0.65)

### Apparatus

The experiment took place in a 1.5 m × 2 m laboratory. Participants cycled on a Mekk Poggio 2.6 Carbon Road Bike with a frame size of 54.5 cm. The bicycle was interfaced with a Tacx Neo trainer system that uses a silent magnetic direct drive to monitor cycle speed and alter pedal resistance (in this study, resistance was fixed to standardise a minimum power output). The Tacx Trainer Version 4 software was used to create the VR environment. The bicycle was set up approximately 1.8 m away from a projector screen that depicted the virtual environment. The virtual environment consisted of a flat topography cityscape with a central park; based upon the American city of Manhattan. Information on cadence, speed, power (watts), slope, and time lapsed were displayed along the bottom of the screen, with the route map displayed on the top right corner of the screen. This study used non-immersive virtual reality.

To obtain heart rate measurements, electrocardiogram (ECG) recordings were taken. Disposable Inmed Blue Sensor T ECG electrodes were placed in a V5, CC5, modified V5R chest configuration [see 39] and sampled via an ADInstruments ML132 Bio Amp connected to an ADInstruments 8/30 PowerLab. The signals from the ECG were acquired and stored on a separate computer using ADInstruments LabPro software.

Auditory feedback was relayed through speakers located at each corner of the laboratory to provide virtual feedback during the cycling task. For each condition, speech feedback was pre-recorded by a female actor and digitized into mp3 files. Feedback statements were provided at 3-minute intervals with silence between feedback statements. There were 10 feedback statements spanning the 30 minutes maximum cycling time. Positive and negative feedback pertained to participants effort, commitment, and performance (e.g., “fantastic / poor effort”, “you are performing better / worse than the other participants”). Neutral feedback referred to objective features of the task such as time and the equipment with no added affective statements (e.g., “the equipment is working as it should be”).

### Exercise performance measures

Exercise performance was measured by persistence, physiological effort, and exertion ratings. Persistence was the amount of time a participant cycled while maintaining at least 70% of their maximal heart rate. The required heart rate level was calculated using the Karvonen formula of: HRR × target% + HRrest [40]. Physiological effort was measured using heart rate. For analyses, changes in effort was calculated as the difference between mean beats per minute in the 30 s prior to the feedback statement and mean beats per minute in the 30 s following the feedback statement. Finally, Rating of Perceived Exertion (RPE) [41] was self-reported on a scale ranging from 6 = *no exertion at all* to 20 = *maximal exertion*. The subjective ratings of exertion have been found to be a relatively accurate measure of actual workload with positive correlations to cardiac intensity (heart rate, *r* = .55) and metabolic intensity (blood lactate concentrations, *r* = .71 [42].

### Psychological experience of exercise measures

#### Intrinsic Motivation Inventory (IMI)

The IMI [43] is a 22-item measure of intrinsic motivation and self-regulation across Interest/Enjoyment, Perceived Competence, Pressure/Tension, and Perceived Choice subscales related to the cycling task used in the current study. Respondents indicated how true the corresponding item is for them on a seven-point scale with the anchors ranging from 1 = *not at all true* to 7 = *very true*. Sample items include *I enjoyed the activity very much* (Interest/Enjoyment), *I think I am pretty good at this activity* (Perceived Competence), *I felt very tense while doing this activity* (Pressure/Tension), and *I believe I had some choice about doing this activity* (Perceived Choice). The current study demonstrated good internal reliability for Interest/Enjoyment subscale (α = .91) and Perceived Competence of (α = .93), and reasonable internal consistency for Pressure/Tension (α = .55) and Perceived Choice (α = .66).

#### Physical Activity Enjoyment Scale (PACES)

The PACES is an 18-item measure of enjoyment associated with exercise [44, 45]. Respondents are asked to evaluate their experiences of the exercise task on a seven-point scale with anchors ranging from 1 = *I enjoy it* to 7 = *I hate it*. Sample items include *I find it pleasurable/ I find it unpleasurable* and *Its not at all stimulating / Its not at all stimulating*. Good internal reliability was demonstrated in the current study (α = .92).

#### Positive and Negative Affect Schedule (PANAS)

PANAS is a 20-item measure of positive and negative state affect [46]. Respondents rated the extent to which they experienced a corresponding emotion during the exercise task on a five-point scale with anchors ranging from 1 = *not at all* to 5 = *extremely*. Sample items include *proud* (positive affect) and *ashamed* (negative affect). Good internal reliability was demonstrated for positive (α = .90) and negative (α = .83) affect in the current study.

### Questionnaires

#### Revised Competitiveness Index (RCI)

The RCI [47] is a 14-item self-report measure designed to capture desire to win, including enjoyment of competition and contentiousness. Respondents are asked to rate their agreement to items on a five-point Likert response scale with the anchors ranging from 1 = *strongly disagree* to 5 = *strongly agree*. Sample items *I get satisfaction from competing with others* and *I try and avoid arguments* (reverse scored). Good internal reliability was demonstrated in the current study (α = .88).

#### International Physical Activity Questionnaire, Long Form (IPAQ-LF)

The IPAQ-LF [48] measured frequency and duration of physical activity and sedentary behaviour across a seven-day period in occupational, transportation, housework, and leisure domains. Respondents indicated whether or not they had undertaken activity in each domain and provided the amount of time spent in the activity. Results were presented as hours spent on average in each category of moderate and vigorous physical activity for descriptive purposes.

#### Feedback rating

A custom-developed scale measured participants perception of the feedback received during the task. This scale provided a manipulation check to ensure that feedback was perceived as intended. Participants were asked to rate their agreement to six items on whether they perceived the feedback to be positive/neutral/negative and whether the feedback made them feel more positive/neutral/negative feedback. Ratings were made on a seven-point scale with the anchors of 1 = *strongly disagree* to 7 = *strongly agree*.

#### Exercise Thoughts Questionnaire (ETQ)

Frequency of exercise avoidant thoughts or cognitive excuses for not exercising was measured with the 25-item ETQ [49] and included for descriptive purposes in the current study. Respondents rate their frequency of excuse making on a five-point scale with anchors ranging from 1 = *not at all* to 5 = *all the time*. Sample items *include I’d rather do something else* and *I have not got time*. Good internal reliability was demonstrated in the current study (α = .92).

### Procedure

Upon arrival to the laboratory, participants provided informed consent, completed the pre-exercise screening tool, and had their height and weight measured. Next, they were fitted with physiological recording equipment and asked to sit quietly for 5 minutes while baseline measurements of heart rate were obtained and recordings checked. The experimenter provided instructions for correct cycling technique and participants cycled for 3 minutes to warm up and familiarise themselves with the equipment and environment. A rest period approximately 10 to up to 20 minutes followed during which the PANAS, ETQ, and IPAQ-LF were completed.

The VR environment and instructions were explained to participants by pre-recorded speech relayed through the speakers. Participants were advised that the experiment was a persistence task and were instructed to cycle for as long as possible at a peddling rate of at least 70 revolutions per minute and while maintaining a minimum heart rate, which corresponded to 70% of HRR, or until 30 minutes has elapsed (whichever is the sooner). The experimenter monitored the peddling rate and heart rate and informed participants when either measure dropped below the required level. Two warnings were provided when performance dropped below the required level and the cycling task was stopped on the third instance. During the task, participants verbally reported their perceived exertion rating at 3-minute time intervals starting as the beginning of cycling. After completing the cycling task, participants completed the PANAS, PACES, RCI, IMI, and Feedback Rating questionnaires. Finally, participants were debriefed, thanked for their time, and awarded their incentive. The study protocol received approval from the institutional human research ethics committee.

### Statistical analysis

The two between-subjects variables were feedback group (positive, neutral, negative) and trait competitiveness level (high, low). A mean split of total RCI scores was used to create high and low trait competitiveness groups. A series of between-subjects factorial analysis of variance (ANOVA) were computed for duration of task persistence and for scores on the self-report scales of PANAS positive state affect, PANAS negative state affect, IMI interest, IMI perceived choice, IMI pressure/tension, IMI perceived competence, and PACES. Homogeneity of variance was violated for scores on PACES, PANAS negative affect, IMI interest, and persistence. Mixed factorial ANOVA’s were computed for physiological effort (heart rate change) and perceived exertion ratings to examine the effects of feedback, trait competitiveness, and the repeated measures factor of time at which effort and exertion ratings were collected. Multiple imputation with the bar procedure was used for missing data (*n* = 122) due to participants ceasing cycling prior to 30 minutes. Extreme scores (as per the statistical analysis) were removed from the dataset for the analysis of perceived exertion (10) and effort (37) producing homogeneity of covariance (set α = .001). Sphericity was violated for perceived exertion χ^2^ (44) = 444.76, *p* < .001, and the Greenhouse Geisser correction applied. Sphericity was violated for effort χ^2^ (44) = 179.04, *p* < .001 and the Greenhouse Geisser correction applied. Post hoc analyses were performed with Tukey’s HSD test to examine main effects and interactions and confirmed with *t*-tests. Statistical Package for the Social Sciences was used for statistical analysis.

## Results

### Exercise performance

Persistence was examined with a 3 × 2 (Feedback × Competitiveness) ANOVA. As shown in Table 2, the time spent cycling was similar across feedback groups and trait competitiveness levels, as shown by all main effects and interactions not reaching significance, all *p* > .05. Perceived exertion and effort were examined with mixed factorial 3 × 2 × 10 (Feedback × Competitiveness × Time) ANOVA’s. Results indicated the main effects of feedback and competitiveness were not statistically significant for perceived exertion or physiological effort, both *p* > .05. The interaction between competitiveness and feedback were also not statistically significant for either measure, both *p* > .05. However, there was a significant main effect of time for perceived exertion, *F* (9, 534) = 109.02, *p* < .001, η_p_^2^ = .47. Follow up pairwise comparisons were conducted to compare between successive time points (e.g., 1 vs. 2, 2 vs. 3). The results indicated that perceived exertion increased over the course of the trial with significant differences (all *p* < .05) between most successive time points (1 vs. 2, 2 vs. 3, 3 vs. 4, 6 vs. 7, 7 vs. 8, and 9 vs. 10). Similarly, there was a significant main effect of time for heart rate change, *F* (6, 523) = 32.58, *p* < .001, η_p_^2^ = .26. Significant differences (*p* < .05) were found to indicate that the change in heart rate was greater (indicating a higher heart rate following the feedback) at time point 1 than time point 2, and time point 7 than time point 8, but was smaller at time point 8 than time point 9.

**Table 2:** Means (standard deviations) for performance measures for low and high trait competitiveness participants in each feedback condition.

	Feedback Condition					
	Positive		Neutral		Negative	
	Low Competitiveness	High Competitiveness	Low Competitiveness	High Competitiveness	Low Competitiveness	High Competitiveness
Persistence (minutes)	11.40 (7.71)	14.20 (10.29)	9.40 (8.51)	13.22 (8.70)	11.89 (10.39)	12.54 (8.11)
Perceived Exertion	15.96 (1.59)	15.66 (1.98)	16.02 (1.95)	16.00 (1.50)	16.13 (2.08)	15.89 (1.44)
Heart Rate Change (bpm)	1.03 (4.76)	0.73 (2.61)	2.06 (0.30)	1.11 (0.12)	1.32 (1.59)	1.35 (0.59)

### Psychological experiences of exercise

Each of the psychological measures were analysed with separate 3 × 2 (Feedback × Competitiveness) ANOVAs. The analyses showed a statistically significant difference between feedback groups for IMI interest scores, *F* (2, 131) = 3.32, *p* = .04, η_p_^2^ = .05. Post hoc analyses indicated that the positive feedback group had higher IMI interest scores than the negative feedback group, *t* (88) = 2.51, *p* = .01, *d* = 0.52. The differences between neutral and negative feedback, and neutral and positive feedback groups were not significant, both *p* > .05. There was no significant main effect of competitiveness or interaction between competitiveness and feedback groups for IMI interest scores, both *p* > .05.

The IMI Competence scores also varied across feedback groups. The statistical analysis yielded a main effect for feedback, *F* (2, 131) = 4.09, *p* = 0.02, η_p_^2^ = .06, and an interaction between competitiveness and feedback, *F* (2, 131) = 3.71, *p* = .03, η_p_^2^ = .05. Follow up analyses yielded a significant difference between positive and negative feedback groups, *t* (38) = 3.39, *p* = .002, *d* = 1.13, and positive and neutral feedback groups *t* (40) = 3.27, *p* = .002, *d* = 1.01, for low competitiveness participants. There was no statistically significant difference between negative and neutral feedback groups for low competitiveness participants, *p* > .05. In addition, there were no significant differences between feedback groups for high competitiveness participants, *p* > .05. There was no significant main effect of competitiveness, *p* >.05. There were no statistically significant main effects or interaction effects for feedback or competitiveness for IMI perceived choice scores or IMI pressure/tension scores, all *p* >.05.

Reported enjoyment tended to higher for the positive feedback group than in the negative feedback group. The statistical analysis confirmed this impression with a main effect of feedback for PACES scores, *F* (2, 131) = 4.39, *p* < .01, η_p_^2^ = .06. Further analyses confirmed the significant difference between positive and negative feedback groups, *t* (88) = 2.83, *p* = .006, *d* = 0.57, as well as between neutral and negative feedback groups, *t* (90) = 3.68, *p* < .001, *d* = 0.59. There was no statistically significant main effect of competitiveness or interaction effect between feedback and competitiveness for PACES scores, both *p*’s > .05.

Finally, there appeared to be no effect of competitiveness or feedback on PANAS subscale scores. The statistical analyses supported this interpretation with no statistically significant main effect or interaction for feedback or competitiveness for PANAS positive or PANAS negative subscale scores, all *p* > .05.

## Discussion

The aim of the present study was to examine the effect of valenced feedback and trait competitiveness on exercise performance measures of persistence, physiological effort, and perceived exertion, and psychological experiences measures of affect, enjoyment, and motivation while participants completed a cycling task within a non-immersive VR environment. It was expected that positive feedback would increase performance and positive psychological experiences in comparison to negative and neutral feedback. It was further predicted that positive feedback would be beneficial to participants low in competitiveness, but that negative feedback might be beneficial for participants high in competitiveness.

Present findings provided partial support for these hypotheses. In particular, positive feedback was shown to enhance intrinsic motivations related to interest compared to negative feedback. Similarly, positive feedback was shown to enhance enjoyment for the cycling task. In addition, participants who were low in trait competitiveness reported greater motivation related to competence when receiving positive feedback. The type of feedback did not appear to influence performance outcomes in either low or high trait competitiveness participants. However, there was an overall effect of feedback on heart rate across the successive time points of the task. Taken together, the present findings suggest that providing virtual feedback as part of a VR cycling task may influence the psychological experiences of exercise but have little effect on actual exercise performance.

The findings for intrinsic motivation differed across the subscales. First, results indicated that feedback has a differential effect on the intrinsic motivation related to interest. Participants in the positive feedback group reported more interest in the cycling task compared to participants in the negative group. This finding is somewhat consistent with previous research that examined overall motivation. For example, Ijsslesteijn et al. [19] found that virtual feedback increased overall exercise motivation with participants reporting more use and value from coaching feedback. Badami, Vaez Mousavi, Wulf, and Namazizadeh [50] also found overall intrinsic motivation was enhanced by positive feedback, although interest motivation was not. Potentially, different types of positive feedback, for example knowledge of results (KR) for good trials used in Badami et al.’s [50] study and affective feedback used in the present study, elicits differential effects for motivation as KR provides information about accuracy [50] and purely affective feedback functions as encouragement or positive reinforcement for effort [51, 52]. Moreover, the current result is consistent with theory that suggests positive feedback can enhance motivation while negative feedback is not conducive to motivation during exercise [10, 11].

As expected, positive feedback resulted in greater motivation related to perceived competence for the cycling task than negative or neutral feedback for participants low in competitiveness. These results are consistent with past research that found higher perceived competence for golf putting when positive feedback was delivered [50]. This study, however, did not explore competitiveness nor the application of VR. Participants lower in competitiveness may have felt more competent due to the positive praise and encouragement delivered during cycling whereas negative feedback may have undermined motivation signalling their effort was not adequate [10–12]. As perceived competence for an exercise task has been found to result in longer times spent exercising and increased adherence to an exercise program [53], it is suggested that positive feedback delivered to individuals who are low in trait competitiveness during VR exercise could result in the achievement of exercise benefits through increased motivation and exercise participation.

The present findings also showed that valenced feedback had an effect on cycling enjoyment, with positive feedback resulting in more enjoyment for the cycling task than negative feedback. Neutral feedback also resulted in more enjoyment for the cycling task when compared to negative feedback. The present findings are in line with a similar VR cycling study which found that receiving positive feedback from a coach resulted in greater enjoyment [54]. One interpretation of this effect is that exercise enjoyment may, in part, be derived from social interaction associated with the feedback. It has previously been demonstrated that enjoyment in a rowing exercise can be increased with the addition of VR environment relative to a non-VR environment [6]. In consideration of this previous research, along with the present findings, there exists the potential that increased enjoyment elicited by exercising in a virtual environment can be enhanced further by the addition of positive feedback to exercisers rather than providing negative feedback.

The current results for affect demonstrated that neither virtual valenced feedback nor competitiveness levels had an effect on negative state affect or positive state affect. These results are inconsistent with previous research that found that positive feedback did enhance positive state affect in runners [16]. However, the methodology employed by Stoate et al. [16] was different to the present study because the effect of positive feedback was inferred from a comparison with a no feedback condition rather than through a comparison with conditions that used negative or neutral feedback. Lewthwaite and Wulf [37] also did not find an effect for feedback on excitement and pleasure using a balance effectiveness task. The feedback used by Lewthwaite and Wulf [37] was framed around social-normative feedback relative to performing better or worse to a group average and this was different to the more general positive or negative feedback used in the present study. These findings and the results of the present study indicate that feedback may not be sufficient to elicit a positive or negative effect on emotion states during physical activity.

The present findings for the performance measures indicated that the manipulation of valenced feedback and trait competitiveness levels did not have an effect on participants cycling performance of persistence, effort, or perceived exertion. This is inconsistent with research that found positive feedback enhanced grip persistence time, perceived effort, perceived exertion [17], and balance [37]. Potentially, positive feedback signalled to participants that their goal had been met and that further effort was not required which is consistent with self-discrepancy, attentional control, and information processing theoretical postulations [13, 55, 56]. This may be particularly the case for the present task where participants were required to maintain a minimum level of performance. Additional increases in performance (i.e., cycling harder) were not rewarded in the context of the cycling task where the main goal was for participants to cycle as long as possible. Furthermore, while past research found enhanced performance benefits for feedback in non-virtual environments [16, 17, 37, 57], no past research exists for performance benefits following valenced feedback in a virtual environment suggesting there may be differences in how feedback functions within VR compared to real environments.

The present research also found that there was no relationship between performance and trait competitiveness. These results are not consistent with previous VR exercise studies which found performance benefits with competitiveness manipulations [30, 31]. These studies, however, introduced a competitive avatar during the experiment creating a specific competitive scenario, whereas the current study did not. Parton and Neumann [58] asked participants to row for 9 minutes in a VR environment. In a condition that participants rowed alone and under the instruction for participants to merely do their best, it was found that high trait competitiveness participants rowed further and maintained a higher power output than low trait competitiveness participants. The contrasting outcomes between the present study and findings reported by Parton and Neumann [58] might reflect the differences in the task constraints. The present task required participants to persist with cycling for as long as possible which potentially was associated with increased pain perception [55], task difficulty [59], and energy output [60, 61] whereas the rowing task by Parton and Neumann [58] explicitly asked participants to do their best over a set (9-minute) period of time which may have been associated with comparatively less pain [62], ease [59], and energy output [60, 61].

### Study limitations and future directions

The results of the current study need to be interpreted with limitations in mind. First, the present sample comprised university students, limiting the generalisability of findings. Future research could draw samples from other populations for examining feedback effects in exercise contexts. This may provide an understanding on how the differential effects of feedback valence translate, for example, to athlete performance [63, 64] as well as regular exercise engagement for the general population [65]. Second, the mean split for trait competitiveness meant that average levels of competitiveness within both groups were included, potentially impacting the ability to find the true influence of valenced feedback on low and high competitiveness for the performance and psychological experiences of exercise outcomes. It is suggested that future research screen for low and high competitiveness for each feedback manipulation. An additional limitation is the variability of exercise duration. In the current study this was one of the main dependent variables however exercise specific factors such as duration may influence psychological factors of exercise (e.g. enjoyment).

Future research could also individualise aspects of the exercise task based on physiological capabilities of the participant rather than standardising these aspects (e.g., bicycle gears) which was done in the present study. This may overcome the influence of exercise intensity on performance and potentially psychological experiences, as this was a limiting factor in the current study with some participants physiologically not able to persist or increase their effort due to cycling within their maximum heart rate zone. Similarly, the length of time spent exercising (cycling in the current study) could be standardised in future research to overcome limitations with interactions (e.g. from enjoyment and affect). Individual differences in psychological factors known to influence performance could also be explored as it was unable to be determined in the present study whether characteristics such as grittiness interacted with feedback. For example, gritty individuals may have persisted for longer despite the valence of feedback [66]. Finally, while the current study used non-immersive VR, level of immersion may influence psychological factors of exercise [see 67]. However, participants using high-immersive VR may have adverse experiences [68].

## Conclusion

Present findings demonstrate that the beneficial effects of positively valenced feedback are not limited to real world exercise environments but can also be observed when exercising in a VR environment. The findings support the suggestion that exercise interventions that are developed for real world contexts can be translated into VR contexts given that VR aims to simulate a real world environment [4]. Moreover, VR-based exercise inherently involves the monitoring of performance parameters (e.g., power output, speed) to translate this effort into virtual movement and the monitoring of physiological signals (e.g., heart rate). VR software can apply this information to provide automated verbal or written feedback. The present findings suggest that this feedback, in particular feedback with a positive valence, will enhance motivational states in exercisers.

In summary, present findings indicated that participants low in competitiveness levels experienced increased motivation related to competence for the VR cycling task when positive feedback was heard. In addition, positive feedback enhanced enjoyment of cycling and interest in cycling. Future research in this area is important to increase the understanding of factors that contribute to exercise performance and other psychological experiences of exercise in order to translate to increased participation in exercise to achieve physiological and psychological health benefits derived from exercise. Designers of VR applications should consider how the social dimension of the VR system [see 69] can be manipulated based on user factors such as traits (e.g. option to exercise in co-active or competitive conditions), as feedback can be manipulated within a VR environment to provide optimal exercise settings, enhancing the psychological aspects of exercise. This may improve VR applications which already have been found to be beneficial for enhancing positive psychological experiences of exercise [5, 6, 70]. Potentially, enhancing performance and psychological experiences of exercise concurrently during exercise could increase participation in exercise or more broadly physical activity. Findings may also inform exercise professionals and psychologists to the effects of feedback manipulations to provide optimal settings for enhancing the experiences of exercise as well as other virtual and reality (non-virtual) based tasks. Given that exercise can be used to prevent and treat health and psychological problems and improve quality of life for people with and without illnesses, the implication is that people should be participating in exercise. Positive feedback delivered during a VR exercise task may assist with achieving the physical and psychological benefits of exercise.
